# Adjunctive Chaihu Guizhi Tang combined with Maxing Shigan Tang and short-term clinical recovery in hospitalized older adults with community-acquired pneumonia: a prospective non-randomized controlled study

**DOI:** 10.3389/fmed.2026.1880550

**Published:** 2026-08-07

**Authors:** Zhaolong Chen, Linling Li, Peixuan Zhu, Suo Li, Yanjing Li

**Affiliations:** Department of Traditional Chinese Medicine, The Thirteenth People’s Hospital of Chongqing/ Chongqing Geriatrics Hospital, Chongqing, China

**Keywords:** Chaihu Guizhi Tang, clinical stability, community-acquired pneumonia, C-reactive protein, Maxing Shigan Tang, older adults, propensity score, traditional Chinese medicine

## Abstract

**Background:**

Older adults hospitalized with community-acquired pneumonia (CAP) often recover slowly despite standard care. Chaihu Guizhi Tang combined with Maxing Shigan Tang (CHGZ + MXSG) is used as adjunctive traditional Chinese medicine for respiratory infections, but prospective evidence in older inpatients is limited. We evaluated its association with short-term clinical recovery.

**Methods:**

In this open-label prospective non-randomized controlled study, 186 older adults hospitalized with CAP received adjunctive CHGZ + MXSG plus standard care (*n* = 93) or standard care alone (*n* = 93) after eligibility confirmation, clinical assessment, and clinician-patient discussion. The primary outcome was day-7 clinical cure. Secondary outcomes included 72-h clinical response, time to clinical stability, antibiotic escalation, treatment failure, length of stay, CRP and symptom-score changes, 28-day readmission and mortality, and prespecified treatment-emergent safety events. Multivariable, expanded covariate-adjusted, propensity-score weighting/matching, and follow-up sensitivity analyses were performed.

**Results:**

Day-7 clinical cure occurred in 74 of 93 participants (79.6%) in the CHGZ + MXSG group and 58 of 93 participants (62.4%) in the standard-care group [unadjusted risk ratio 1.28, 95% confidence interval (CI) 1.06 to 1.54; *P* = 0.010). The CHGZ + MXSG group also had earlier clinical response at 72 h, less antibiotic escalation, shorter mean time to clinical stability, shorter hospital stay, greater CRP reduction by day 5, and greater symptom-score reduction by day 7. In the multivariable-adjusted model, CHGZ + MXSG was associated with higher odds of day-7 clinical cure [odds ratio (OR) 2.37, 95% CI 1.18–4.90], earlier clinical stability [hazard ratio (HR) 1.73, 95% CI 1.27–2.35], and shorter hospital stay (beta −0.99 days, 95% CI −1.49 to −0.49). These findings were directionally consistent across expanded covariate-adjusted analyses, stabilized inverse probability of treatment weighting, trimmed weighting, overlap weighting, 1:1 propensity-score matching, and follow-up sensitivity analyses.

**Conclusion:**

Among hospitalized older adults with CAP, adjunctive CHGZ + MXSG plus standard care was associated with faster short-term clinical recovery and no descriptive excess of the prespecified safety events. Because treatment allocation was not randomized and outcome assessment was open-label, these findings should be interpreted as adjusted associations rather than definitive causal effects and should be evaluated in adequately powered randomized or pragmatic trials.

## 1 Introduction

Community-acquired pneumonia (CAP) remains one of the most important acute infectious diseases in adult medicine, with a substantial burden of hospitalization, functional decline, and death. Contemporary CAP guidelines emphasize early diagnosis, severity assessment, appropriate antimicrobial therapy, and reassessment as clinical and microbiological information becomes available (1, 2). The burden is particularly concentrated in older adults, in whom multimorbidity, frailty, atypical presentations, and reduced physiological reserve can complicate diagnosis and recovery (3–5). Recent global estimates continue to show that lower respiratory infections remain a major source of morbidity and mortality across age groups, despite improvements in antimicrobial and supportive care (6).

Clinical severity scores such as the Pneumonia Severity Index (PSI) and CURB-65 help clinicians stratify risk at presentation and identify patients who may need inpatient care or closer monitoring (7, 8). In older hospitalized patients, however, the most relevant bedside question is often not only survival, but the speed and completeness of clinical recovery. Clinical stability has therefore become a practical outcome in CAP research because it captures resolution of fever, hemodynamic instability, respiratory compromise, and impaired oral intake, and it informs discharge decisions and antibiotic duration (9–11).

Inflammation is another important dimension of CAP recovery. C-reactive protein (CRP) is not a substitute for clinical judgment, but serial CRP measurements have been associated with severity, complications, and treatment failure in hospitalized CAP (12–14). The concept that excessive or persistent inflammation may contribute to delayed recovery is also reflected in trials of adjunctive anti-inflammatory therapy for selected patients with severe CAP (15, 16). These data do not imply that every anti-inflammatory intervention is beneficial, but they provide a rationale for studying adjunctive approaches that may influence symptom resolution and inflammatory trajectories.

Traditional Chinese medicine (TCM) is frequently used as adjunctive therapy for respiratory infections in China. For CAP, evidence-based guidance for Chinese patent medicines has emphasized both potential benefit and the need for more rigorous clinical evaluation (17). Maxing Shigan Tang and related prescriptions have been investigated in respiratory infections and CAP, with systematic reviews suggesting possible clinical benefit when added to conventional therapy, although the quality and generalizability of available evidence remain variable (18, 19). A randomized multicenter study of a related Maxing Ganshi formula in pediatric CAP also reported improvement in clinical symptoms and fever resolution, but evidence in older hospitalized adults remains much less developed (20).

Chaihu Guizhi Tang combined with Maxing Shigan Tang (CHGZ + MXSG) is used clinically as an adjunctive regimen in patients with respiratory infections. The older inpatient CAP population is a particularly relevant group in which to study this approach, because treatment goals include not only microbiological control but also faster stabilization, reduced need for treatment escalation, preservation of function, and avoidance of adverse events. We therefore conducted a prospective non-randomized controlled study to evaluate whether adjunctive CHGZ + MXSG plus standard care was associated with improved short-term clinical recovery compared with standard care alone in hospitalized older adults with CAP.

From a clinical and pharmacological perspective, the combined regimen was selected to target both alternating exterior-interior manifestations and lung heat-related cough or wheezing during the acute recovery period. Evidence from related Maxing Shigan-based regimens cannot be directly extrapolated to the combined CHGZ + MXSG regimen or to multimorbid older inpatients, in whom frailty, polypharmacy, atypical presentation, and adverse-event vulnerability may modify both recovery and safety. The specific knowledge gap was therefore whether adjunctive CHGZ + MXSG, when added to routine inpatient CAP care, was associated with faster short-term clinical recovery without a descriptive increase in prespecified treatment-emergent safety events.

## 2 Materials and methods

### 2.1 Study design and reporting

This was a prospective non-randomized controlled study of hospitalized older adults with CAP. Participants were enrolled after eligibility screening and were assigned to one of two treatment strategies: adjunctive CHGZ + MXSG plus standard care or standard care alone. Because treatment allocation was not randomized, all analyses were planned and interpreted as comparisons of adjusted associations rather than as randomized treatment effects. The manuscript was prepared with attention to reporting principles for observational studies and non-randomized intervention evaluations (21, 22). The study was open-label; participants, treating clinicians, and outcome assessors were aware of treatment allocation. The study was not registered in a public clinical trial registry before enrollment.

The study was conducted in accordance with the Declaration of Helsinki. The study was approved by the Ethics Committee of the Chongqing Thirteenth People’s Hospital (approval number: cqtp251108). Written informed consent was obtained before enrollment according to the approved protocol.

### 2.2 Participants

Eligible participants were hospitalized adults aged 65 years or older with CAP and available baseline treatment-allocation and outcome data. CAP was diagnosed by treating clinicians on the basis of compatible acute respiratory symptoms, clinical assessment, and radiographic evidence of pulmonary infection, consistent with standard CAP diagnostic practice (1). Patients who declined participation, did not meet the inclusion criteria, or had other protocol-defined reasons for exclusion were not enrolled. Key exclusion criteria included suspected aspiration pneumonia as the primary diagnosis, direct intensive care unit admission at enrollment, severe immunosuppression, decompensated organ failure judged by the treating team to dominate the clinical course, active use of another herbal formula for respiratory infection during the study period, and inability to complete the day-7 clinical assessment according to the protocol.

The full analysis population included all 186 enrolled participants with baseline treatment allocation and day-7 clinical cure data. The per-protocol population included 179 participants who completed the assigned treatment protocol and did not withdraw from follow-up; participant follow-up and evaluable populations are summarized in Supplementary Table S1.

### 2.3 Treatment strategies

Participants in the adjunctive-treatment group received the study-defined Chaihu Guizhi Tang combined with Maxing Shigan Tang (CHGZ + MXSG) regimen in addition to standard care. The herbal regimen was developed according to the classical six-channel differentiation framework of Shang Han Lun and the institutional protocol for older adults with community-acquired pneumonia. The core prescription combined Chaihu Guizhi Tang, traditionally used to harmonize the lesser yang and regulate the exterior–interior relationship, with Maxing Shigan Tang, traditionally used to diffuse lung qi, clear heat, and relieve cough and wheezing. The core components of Chaihu Guizhi Tang included Chaihu, Huangqin, Guizhi, Shaoyao, Renshen, Banxia, Zhigancao, Shengjiang, and Dazao; Maxing Shigan Tang included Mahuang, Xingren, Shigao, and Gancao. Minor modifications were permitted according to the patient’s presenting six-channel pattern and clinical manifestations, but the number of additional or substituted herbs was restricted by the study protocol to avoid excessive heterogeneity in the intervention.

The regimen was administered as a hospital-prepared oral decoction under the institutional TCM protocol, generally in two divided daily doses during the acute inpatient treatment window. Minor symptom-pattern modifications were permitted only within the protocol-defined framework and were intended to avoid excessive intervention heterogeneity; concurrent use of other herbal formulas for respiratory infection was not allowed during the study period.

Participants in the comparison group received standard care alone. Standard care consisted of routine inpatient management for community-acquired pneumonia at the study hospital, including antimicrobial treatment, oxygen therapy when indicated, antipyretic treatment, airway management, fluid and nutritional support, and monitoring of clinical and laboratory parameters according to the treating physician’s assessment. Escalation or adjustment of antimicrobial therapy was allowed when clinically necessary, based on disease progression, microbiological information, or inadequate clinical response. The same institutional CAP care pathway was used for both groups. Discharge was considered after sustained clinical stability, improving respiratory status, ability to continue oral treatment when appropriate, and absence of a need for treatment escalation, while final discharge decisions remained part of routine clinical care.

Treatment assignment followed a prospective non-randomized, open-label strategy and was not generated by a random sequence, calendar period, or ward-based schedule. After eligibility confirmation, treatment allocation was made through clinician-patient discussion in the context of routine care, clinical assessment, and TCM syndrome differentiation. This process may introduce selection bias and confounding by indication; therefore, all analyses were interpreted as adjusted associations rather than causal treatment effects. All participants were managed under the same inpatient care pathway for community-acquired pneumonia, and outcome assessment was based on prespecified clinical, laboratory, and safety endpoints.

### 2.4 Outcomes

The main clinical outcome was clinical cure at day 7, defined as clear improvement or resolution of pneumonia-related symptoms, no persistent fever or hemodynamic instability, no need for antibiotic escalation or rescue escalation because of inadequate clinical response, and no death or readmission before the day-7 assessment. Borderline cases were classified as not clinically cured if any prespecified criterion for treatment failure or inadequate recovery was present. Early clinical response at 72 h was analyzed as a secondary binary outcome and required early symptom improvement without clinical deterioration or treatment escalation.

Time to clinical stability was defined as the interval from treatment assignment to the first day on which prespecified stability criteria were met. The stability framework was based on conventional CAP clinical-stability constructs involving temperature, respiratory status, hemodynamic stability, oxygenation, and clinical ability to continue recovery without escalation (9, 11). Additional secondary outcomes included antibiotic escalation, treatment failure, 28-day readmission, 28-day all-cause mortality, hospital length of stay, decrease in CRP from baseline to day 5, and reduction in a composite symptom score from baseline to day 7. Treatment-emergent safety outcomes included gastrointestinal adverse events, alanine aminotransferase (ALT) elevation events, and rash. Clinical stability required fulfillment of prespecified bedside criteria, including temperature no higher than 37.8 °C, systolic blood pressure at least 90 mmHg without new hemodynamic support, respiratory rate no higher than 24 breaths/min, stable or improving oxygenation, and the ability to continue recovery without immediate escalation of antimicrobial or respiratory support. Treatment failure was defined as lack of clinical improvement requiring escalation of therapy, clinical deterioration, death, or readmission related to pneumonia during follow-up.

The composite symptom score was an institutional ordinal symptom measure recorded by clinical staff, with higher scores indicating greater symptom burden; cough and fatigue were prespecified component items and were summarized separately. Pathogen categories were assigned from available routine microbiological information, including sputum or blood culture and respiratory viral or atypical pathogen testing when clinically obtained. Testing followed routine inpatient practice and was interpreted using the same diagnostic framework in both treatment groups. Outcome assessment was performed according to prespecified criteria but was not blinded to treatment allocation.

### 2.5 Statistical analysis

Continuous variables were summarized as mean with standard deviation or median with interquartile range, as appropriate. Categorical variables were summarized as counts and percentages. Baseline balance was evaluated primarily with standardized mean differences (SMDs), because *P*-values are not an appropriate substitute for assessing imbalance in non-randomized treatment comparisons (23). Descriptive *P*-values were provided for transparency.

Unadjusted binary outcomes were summarized using risk ratios with 95% confidence intervals (CIs). Continuous outcomes were summarized using mean differences with 95% CIs. Time to clinical stability was analyzed using Kaplan-Meier curves, the log-rank test, and Cox proportional hazards models. For CRP trajectories, estimated marginal means and 95% CIs were derived from a linear mixed-effects model including treatment group, time, and the group-by-time interaction, with a participant-level random intercept.

For key clinical outcomes, the prespecified multivariable-adjusted models included age, sex, CURB-65 score, chronic obstructive pulmonary disease (COPD), and baseline CRP. Because several baseline variables showed potentially relevant imbalance, additional sensitivity analyses used an expanded geriatric-confounder model including BMI, frailty score, PSI score, respiratory rate, baseline SpO2, congestive heart failure, diabetes, chronic kidney disease, PCT, and pathogen category where appropriate. Logistic regression was used for day-7 clinical cure, Cox proportional hazards regression was used for time to clinical stability, and linear regression was used for hospital length of stay.

To address measured confounding due to non-randomized treatment allocation, propensity-score sensitivity analyses were performed. The propensity score was estimated using baseline demographic characteristics, clinical severity indicators, comorbidities, laboratory markers, and pathogen category variables available before treatment allocation. Stabilized inverse probability of treatment weighting (IPTW), trimmed IPTW with weights truncated at the 1st and 99th percentiles, overlap weighting, and 1:1 nearest-neighbor propensity-score matching without replacement using a caliper of 0.20 standard deviations of the logit propensity score were applied (24–27). Covariate balance before and after weighting or matching was evaluated using SMDs, and SMD values below 0.10 were considered to indicate acceptable balance.

Exploratory subgroup analyses evaluated day-7 clinical cure by age group, sex, COPD status, PSI risk class, and CURB-65 category. Interaction *P* values were obtained from models including treatment-by-subgroup interaction terms. Subgroup analyses were exploratory and were not adjusted for multiplicity. Statistical analyses were performed using R software.

No formal prospective sample-size calculation was performed. The precision of the observed primary endpoint and the detectable risk difference under the enrolled sample size were summarized descriptively. Missingness was summarized by treatment group; the day-7 clinical cure endpoint had complete data, and sensitivity analyses evaluated whether assumptions about incomplete follow-up or protocol completion changed the direction of the primary endpoint.

## 3 Results

### 3.1 Study flow and baseline characteristics

**FIGURE 1:**
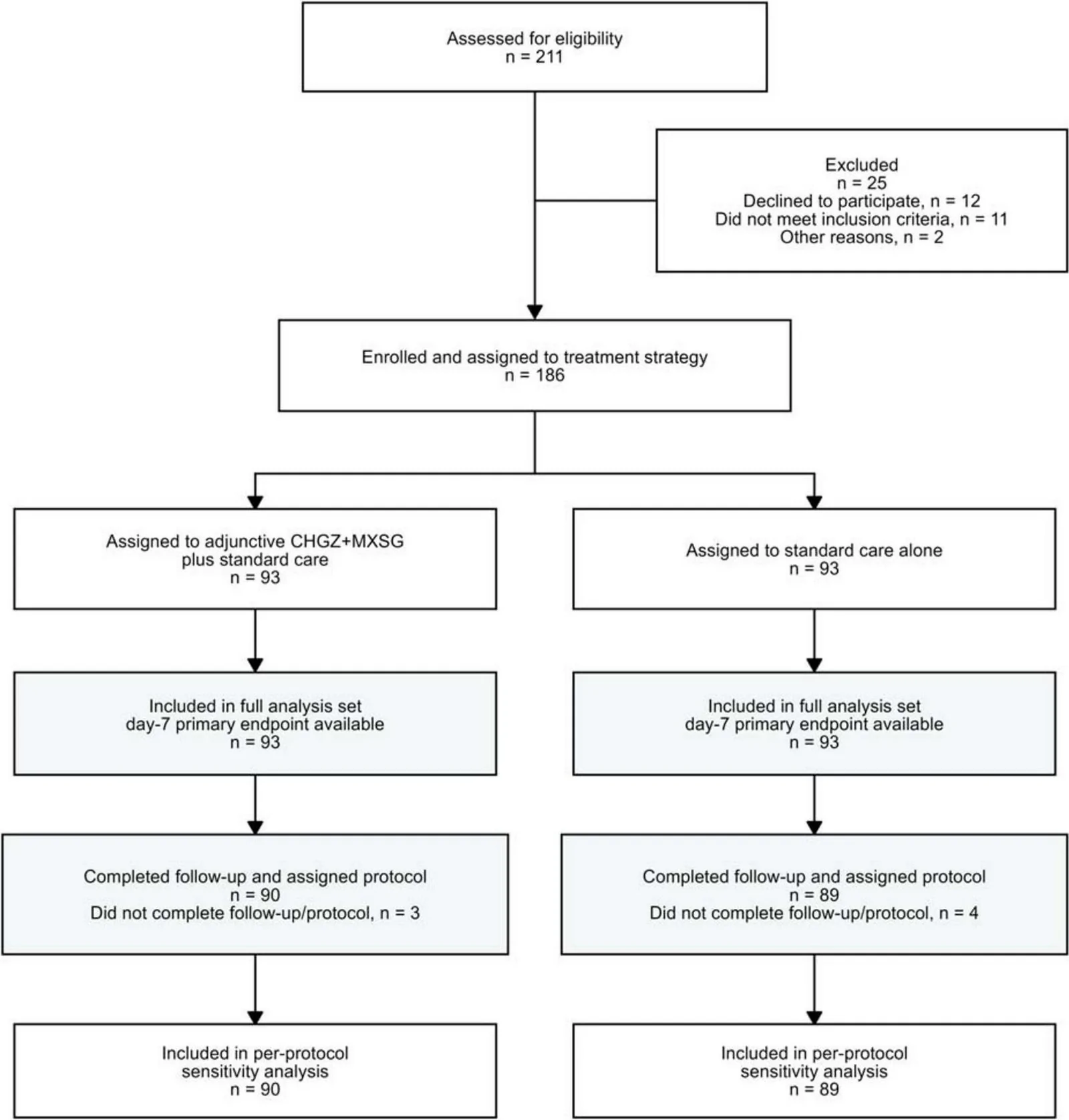
Study flow diagram. Among 211 hospitalized older adults with community-acquired pneumonia assessed for eligibility, 25 were excluded, including 12 who declined to participate, 11 who did not meet the inclusion criteria, and 2 for other reasons. A total of 186 participants were enrolled and assigned to adjunctive CHGZ + MXSG plus standard care or standard care alone, with 93 participants in each group. All enrolled participants had available day-7 clinical cure data and were included in the full analysis set according to their initial treatment strategy. Complete follow-up and assigned treatment protocol were achieved by 90 participants in the CHGZ + MXSG group and 89 participants in the standard-care group; these participants were included in the per-protocol sensitivity analysis. CHGZ + MXSG, Chaihu Guizhi Tang combined with Maxing Shigan Tang.

Among 211 hospitalized older adults assessed for eligibility, 25 were excluded: 12 declined to participate, 11 did not meet inclusion criteria, and 2 were excluded for other reasons. All 186 enrolled participants had available day-7 clinical cure data and were included in the full analysis set according to their initial treatment strategy. Complete follow-up and assigned treatment protocol were achieved by 90 participants in the CHGZ + MXSG group and 89 participants in the standard-care group, and these participants formed the per-protocol sensitivity-analysis population (Figure 1). Complete day-7 clinical cure data were available for all enrolled participants; 179 participants completed follow-up and the assigned treatment protocol, forming the per-protocol population (Supplementary Tables S1,S2).

Baseline characteristics are summarized in Table 1. The mean age was 74.4 ± 5.8 years in the CHGZ + MXSG group and 73.7 ± 5.8 years in the standard-care group. Male participants accounted for 58.1% and 60.2% of the two groups, respectively. Baseline disease severity was similar by CURB-65 score (1.7 ± 0.8 vs. 1.7 ± 0.7) and PSI score (93.9 ± 17.9 vs. 92.9 ± 14.9). Median baseline CRP was 93.2 mg/L [interquartile range (IQR), 71.2–120.5] in the CHGZ + MXSG group and 96.8 mg/L (IQR, 71.5–120.1) in the standard-care group.

**TABLE 1:** Baseline characteristics of the full analysis set in the prospective non-randomized controlled study.

Characteristic	CHGZ + MXSG (*n* = 93)	Standard care (*n* = 93)	SMD	Descriptive *P*-value
Age, years	74.4 ± 5.8	73.7 ± 5.8	0.135	0.359
Body mass index, kg/m2	23.8 ± 3.1	23.7 ± 3.2	0.009	0.951
Frailty score	3.7 ± 1.2	3.5 ± 1.2	0.204	0.166
CURB-65 score	1.7 ± 0.8	1.7 ± 0.7	0.000	1.000
PSI score	93.9 ± 17.9	92.9 ± 14.9	0.064	0.662
Temperature at baseline, °C	38.4 ± 0.5	38.3 ± 0.4	0.057	0.696
Respiratory rate at baseline, breaths/min	24.9 ± 3.3	24.2 ± 3.3	0.217	0.142
Systolic blood pressure at baseline, mmHg	121.9 ± 13.1	120.9 ± 14.3	0.076	0.605
SpO2 at baseline, %	91.9 ± 2.7	91.9 ± 2.6	0.008	0.956
WBC at baseline, × 10^9^/L	12.8 ± 2.9	12.6 ± 3.1	0.069	0.639
Neutrophil count at baseline, × 10^9^/L	9.7 ± 3.2	9.4 ± 2.7	0.108	0.461
CRP at baseline, mg/L	93.2 (71.2, 120.5)	96.8 (71.5, 120.1)	0.024	0.868
PCT at baseline, ng/mL	0.4 (0.3, 0.6)	0.4 (0.2, 0.7)	0.186	0.207
BUN at baseline, mmol/L	7.5 (5.9, 9.0)	7.5 (6.0, 9.2)	0.029	0.843
ALT at baseline, U/L	29.0 (24.0, 39.0)	31.0 (26.0, 38.0)	0.051	0.730
Male sex	54 (58.1%)	56 (60.2%)	0.044	0.765
Current smoker	19 (20.4%)	20 (21.5%)	0.026	0.857
COPD	39 (41.9%)	49 (52.7%)	0.215	0.142
Congestive heart failure	19 (20.4%)	11 (11.8%)	0.234	0.111
Diabetes mellitus	21 (22.6%)	28 (30.1%)	0.171	0.244
Chronic kidney disease	16 (17.2%)	10 (10.8%)	0.186	0.205
PSI risk class, n (%)			0.021	0.999
II	7 (7.5%)	7 (7.5%)		
III	32 (34.4%)	31 (33.3%)		
IV	53 (57.0%)	54 (58.1%)		
V	1 (1.1%)	1 (1.1%)		
Pathogen category, n (%)			0.120	0.642
Bacterial pathogen	44 (47.3%)	38 (40.9%)		
Atypical pathogen	12 (12.9%)	11 (11.8%)		
Viral/mixed pathogen	17 (18.3%)	24 (25.8%)		
No pathogen identified	20 (21.5%)	20 (21.5%)		

Data are presented as mean ± SD for approximately normally distributed continuous variables, median (IQR) for skewed continuous variables, and n (%) for categorical variables. Because treatment allocation was non-randomized, baseline balance should be interpreted using standardized mean differences rather than formal significance testing; descriptive *P* values are provided for transparency. SMD > = 0.10 may indicate a potentially relevant imbalance. SMD, standardized mean difference; PSI, Pneumonia Severity Index; COPD, chronic obstructive pulmonary disease; CRP, C-reactive protein; PCT, procalcitonin; BUN, blood urea nitrogen; ALT, alanine aminotransferase; CHGZ + MXSG, Chaihu Guizhi Tang combined with Maxing Shigan Tang.

Because treatment allocation was non-randomized, baseline balance was interpreted using SMDs. Potentially relevant imbalances were observed for frailty score (SMD 0.204), respiratory rate (SMD 0.217), COPD (SMD 0.215), congestive heart failure (SMD 0.234), diabetes mellitus (SMD 0.171), chronic kidney disease (SMD 0.186), and PCT (SMD 0.186). These imbalances supported the use of multivariable and propensity-score adjusted analyses.

### 3.2 Unadjusted clinical outcomes

Unadjusted clinical outcomes are shown in Table 2. Day-7 clinical cure occurred in 74 of 93 participants (79.6%) in the CHGZ + MXSG group and 58 of 93 participants (62.4%) in the standard-care group [risk ratio (RR) 1.28, 95% CI 1.06–1.54; *P* = 0.010]. Early clinical response at 72 h was also more frequent in the CHGZ + MXSG group [67 of 93 (72.0%) vs. 40 of 93 (43.0%); RR 1.68, 95% CI 1.28–2.19; *P* < 0.001].

**TABLE 2:** Unadjusted primary and secondary clinical outcomes in the full analysis set.

Outcome	CHGZ + MXSG (*n* = 93)	Standard care (*n* = 93)	Unadjusted effect estimate (95% CI)	Descriptive *P*-value
Clinical cure at day 7	74 (79.6%)	58 (62.4%)	RR 1.28 (1.06–1.54)	0.010
Early clinical response at 72 h	67 (72.0%)	40 (43.0%)	RR 1.68 (1.28–2.19)	< 0.001
Antibiotic escalation	5 (5.4%)	17 (18.3%)	RR 0.29 (0.11–0.76)	0.006
Treatment failure	16 (17.2%)	22 (23.7%)	RR 0.73 (0.41–1.29)	0.275
Readmission within 28 days	2 (2.2%)	6 (6.5%)	RR 0.33 (0.07–1.61)	0.278
28-day all-cause mortality	1 (1.1%)	2 (2.2%)	RR 0.50 (0.05–5.42)	1.000
Time to clinical stability, days	5.0 ± 1.3	5.8 ± 1.4	MD −0.84 (−1.23 to −0.45)	< 0.001
Hospital length of stay, days	8.8 ± 1.6	9.9 ± 1.9	MD −1.10 (−1.61 to −0.59)	< 0.001
Decrease in CRP from baseline to day 5, mg/L	59.7 ± 14.6	54.7 ± 15.1	MD 5.00 (0.64–9.37)	0.025
Reduction in composite symptom score from baseline to day 7	12.2 ± 2.9	11.0 ± 2.8	MD 1.26 (0.43–2.09)	0.003

Data are presented as n (%) for binary outcomes and mean ± SD for continuous outcomes unless otherwise specified. Estimates are unadjusted and compare CHGZ + MXSG with standard care. For binary outcomes, effect estimates are risk ratios; for continuous outcomes, effect estimates are mean differences. Negative mean differences indicate shorter time to clinical stability or shorter hospitalization in the CHGZ + MXSG group. For CRP decrease and composite symptom score reduction, larger values indicate greater improvement. Adjusted and propensity-score sensitivity analyses are summarized in Table 4. RR, risk ratio; MD, mean difference; CI, confidence interval; CRP, C-reactive protein; CHGZ + MXSG, Chaihu Guizhi Tang combined with Maxing Shigan Tang.

Antibiotic escalation occurred in 5 participants (5.4%) in the CHGZ + MXSG group and 17 participants (18.3%) in the standard-care group (RR 0.29, 95% CI 0.11–0.76; *P* = 0.006). Treatment failure occurred in 16 participants (17.2%) and 22 participants (23.7%), respectively (RR 0.73, 95% CI 0.41–1.29; *P* = 0.275). Readmission within 28 days and 28-day all-cause mortality were uncommon and did not differ materially between groups in descriptive comparisons.

### 3.3 Time to clinical stability, length of stay, and CRP trajectory

**FIGURE 2:**
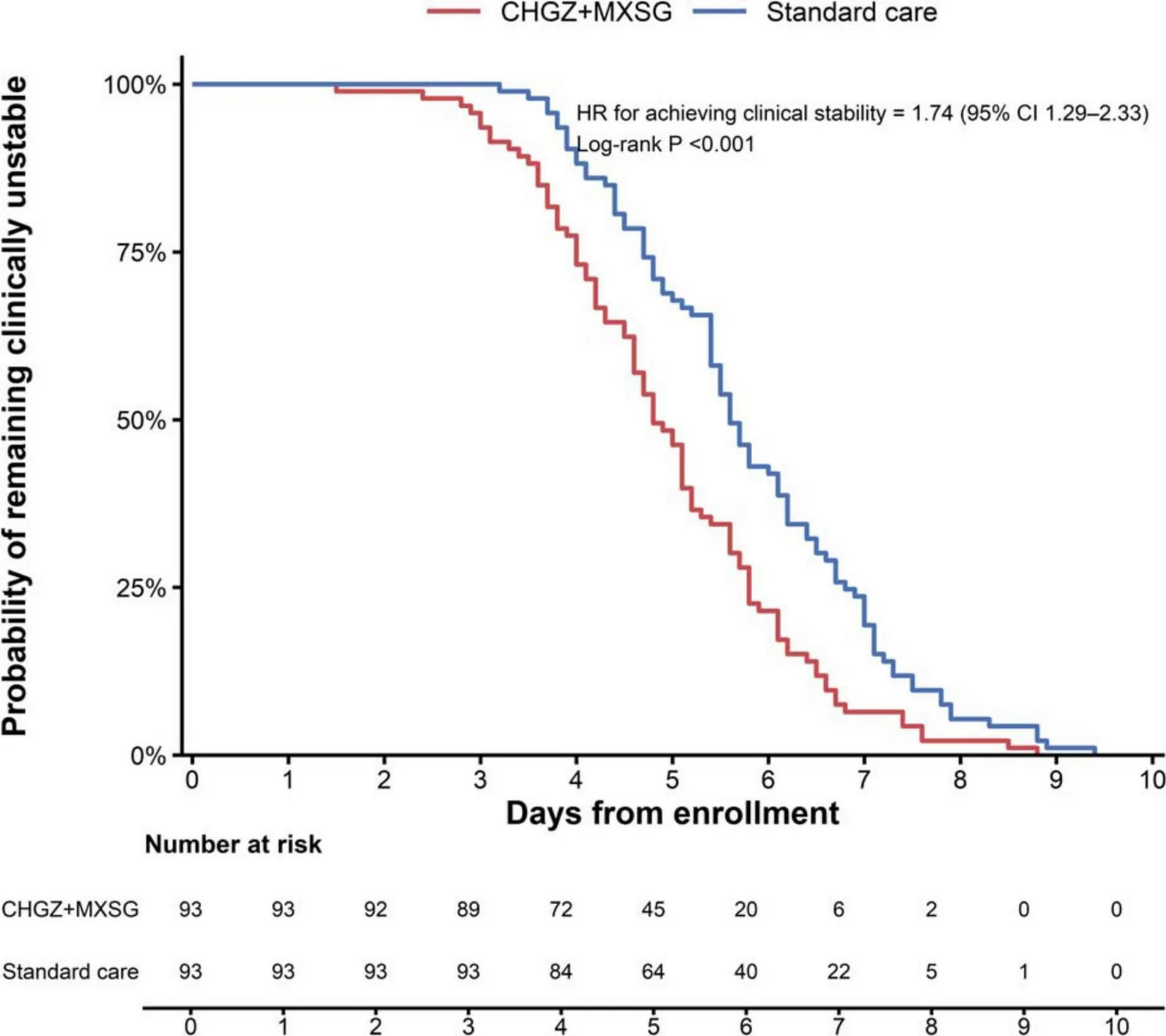
Time to clinical stability in the full analysis set. Kaplan-Meier curves show the probability of remaining clinically unstable after treatment assignment. Lower curves indicate earlier achievement of clinical stability. The hazard ratio was estimated using an unadjusted Cox proportional hazards model, with a hazard ratio greater than 1 indicating faster achievement of clinical stability. The *P*-value was derived from the log-rank test. Numbers at risk are shown below the plot. CHGZ + MXSG, Chaihu Guizhi Tang combined with Maxing Shigan Tang; CI, confidence interval.

The mean time to clinical stability was 5.0 ± 1.3 days in the CHGZ + MXSG group and 5.8 ± 1.4 days in the standard-care group (mean difference −0.84 days, 95% CI −1.23 to −0.45; *P* < 0.001). Kaplan-Meier curves showed earlier achievement of clinical stability in the CHGZ + MXSG group; the unadjusted hazard ratio for achieving clinical stability was 1.74 (95% CI 1.29–2.33), with a log-rank P < 0.001 (Figure 2).

**FIGURE 3:**
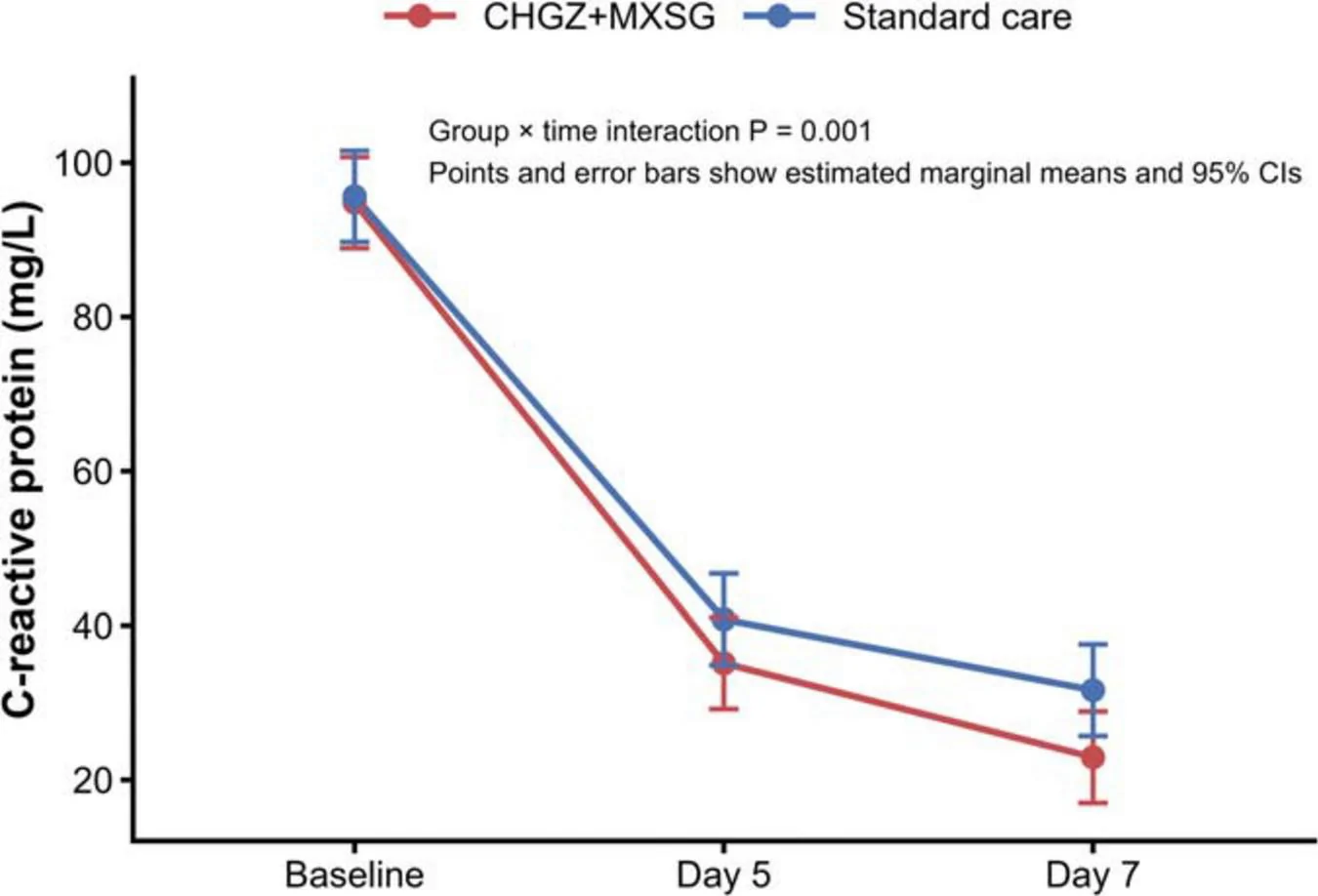
Trajectory of C-reactive protein during follow-up in the full analysis set. Points represent estimated marginal means and error bars represent 95% confidence intervals derived from a linear mixed-effects model including treatment group, time, and the group-by-time interaction, with a participant-level random intercept. C-reactive protein decreased over time in both groups, with a greater reduction in the CHGZ + MXSG group. The *P*-value shown in the panel corresponds to the group-by-time interaction. CHGZ + MXSG, Chaihu Guizhi Tang combined with Maxing Shigan Tang; CI, confidence interval.

Hospital length of stay was shorter in the CHGZ + MXSG group than in the standard-care group (8.8 ± 1.6 vs. 9.9 ± 1.9 days; mean difference −1.10 days, 95% CI −1.61 to −0.59; *P* < 0.001). The decrease in CRP from baseline to day 5 was greater in the CHGZ + MXSG group (59.7 ± 14.6 vs. 54.7 ± 15.1 mg/L; mean difference 5.00 mg/L, 95% CI 0.64–9.37; *P* = 0.025). In the repeated-measures analysis, CRP declined over time in both groups, with a greater reduction in the CHGZ + MXSG group (group-by-time interaction *P* = 0.001; Figure 3). Reduction in the composite symptom score from baseline to day 7 was also greater in the CHGZ + MXSG group (12.2 ± 2.9 vs. 11.0 ± 2.8; mean difference 1.26, 95% CI 0.43–2.09; *P* = 0.003). In component analyses, reductions in cough score and fatigue score also favored CHGZ + MXSG, supporting the direction of the composite symptom-score result (Supplementary Table S6).

### 3.4 Adjusted and propensity-score sensitivity analyses

Adjusted and propensity-score sensitivity analyses are summarized in Table 3 and Supplementary Table S4. In the multivariable-adjusted model, adjunctive CHGZ + MXSG was associated with higher odds of day-7 clinical cure (OR 2.37, 95% CI 1.18–4.90; *P* = 0.017), earlier clinical stability (HR 1.73, 95% CI 1.27–2.35; *P* < 0.001), and shorter hospital stay (beta −0.99 days, 95% CI −1.49 to −0.49; *P* < 0.001). In expanded geriatric-confounder models, the direction of association remained consistent for day-7 clinical cure (OR 2.79, 95% CI 1.26–6.21), clinical stability (HR 1.98, 95% CI 1.42–2.77), and hospital length of stay (beta −1.00 days, 95% CI −1.50 to −0.51).

**TABLE 3:** Adjusted and propensity-score sensitivity analyses for key clinical outcomes in the full analysis set.

Analysis	Clinical cure at day 7 OR (95% CI); *P* value	Time to clinical stability HR (95% CI); *P* value	Hospital length of stay beta (95% CI); *P* value
Multivariable-adjusted model	2.37 (1.18–4.90); *P* = 0.017	1.73 (1.27–2.35); *P* < 0.001	−0.99 (−1.49 to −0.49); *P* < 0.001
Stabilized IPTW	2.36 (1.18–4.75); *P* = 0.016	1.73 (1.27–2.34); *P* < 0.001	−1.08 (−1.65 to −0.51); *P* < 0.001
Trimmed IPTW	2.39 (1.19–4.79); *P* = 0.014	1.74 (1.29–2.36); *P* < 0.001	−1.08 (−1.65 to −0.52); *P* < 0.001
Overlap weighting	2.48 (1.23–5.00); *P* = 0.011	1.78 (1.31–2.42); *P* < 0.001	−1.07 (−1.63 to −0.52); *P* < 0.001
1:1 propensity-score matching	3.82 (1.85–7.89); *P* < 0.001	1.89 (1.33–2.69); *P* < 0.001	−1.11 (−1.79 to −0.44); *P* = 0.001
Per-protocol sensitivity analysis	3.01 (1.45–6.52); *P* = 0.004	–	–

All estimates compare adjunctive CHGZ + MXSG plus standard care with standard care alone. The multivariable-adjusted models included age, sex, CURB-65 score, COPD, and baseline CRP. The propensity-score model included baseline demographic characteristics, clinical severity indicators, comorbidities, laboratory markers, and pathogen-category variables available before treatment allocation. Stabilized IPTW estimated the average treatment effect in the full analysis set; trimmed IPTW truncated weights at the 1st and 99th percentiles; overlap weighting targeted the clinical equipoise population; and propensity-score matching used 1:1 nearest-neighbor matching without replacement with a caliper of 0.20 SD of the logit propensity score. OR > 1 favors adjunctive CHGZ + MXSG for day-7 clinical cure; HR > 1 indicates earlier achievement of clinical stability; negative beta indicates shorter hospital length of stay. Because treatment allocation was not randomized, these estimates should be interpreted as adjusted associations rather than definitive causal effects. CHGZ + MXSG, Chaihu Guizhi Tang combined with Maxing Shigan Tang; IPTW, inverse probability of treatment weighting; PSM, propensity-score matching; OR, odds ratio; HR, hazard ratio; CRP, C-reactive protein; COPD, chronic obstructive pulmonary disease.

The direction and approximate magnitude of these associations were consistent across stabilized IPTW, trimmed IPTW, overlap weighting, and 1:1 propensity-score matching. Across these propensity-score analyses, ORs for day-7 clinical cure ranged from 2.36 to 3.82, HRs for clinical stability ranged from 1.73 to 1.89, and adjusted differences in hospital length of stay ranged from −1.07 to −1.11 days. Propensity-score diagnostics showed improved covariate balance after weighting, with all covariates achieving SMD values below 0.10 after stabilized IPTW, trimmed IPTW, and overlap weighting (Supplementary Table S5). The per-protocol sensitivity analysis for day-7 clinical cure was directionally consistent with the full-analysis-set result (OR 3.01, 95% CI 1.45–6.52; *P* = 0.004), and follow-up assumption analyses did not change the direction of the primary endpoint (Supplementary Table S3).

### 3.5 Subgroup and safety analyses

**FIGURE 4:**
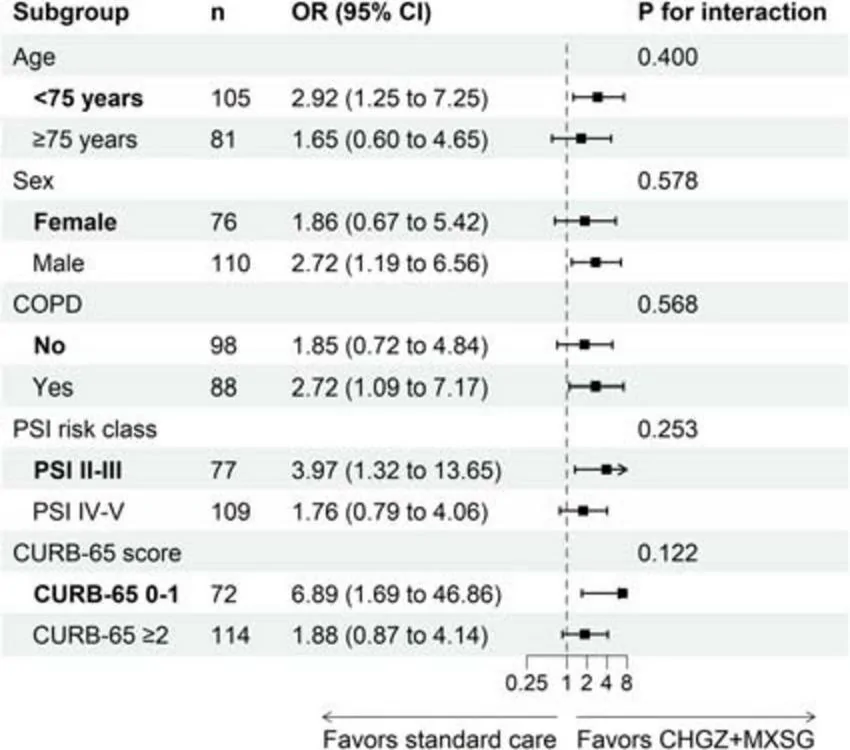
Exploratory subgroup analyses for day-7 clinical cure. Odds ratios and 95% confidence intervals compare adjunctive CHGZ + MXSG plus standard care with standard care alone for day-7 clinical cure. Odds ratios greater than 1 favor adjunctive CHGZ + MXSG. The dashed vertical line indicates no treatment effect. *P*-values for interaction were obtained from models including treatment-by-subgroup interaction terms. These analyses were exploratory and were not adjusted for multiplicity. CHGZ + MXSG, Chaihu Guizhi Tang combined with Maxing Shigan Tang; OR, odds ratio; CI, confidence interval; COPD, chronic obstructive pulmonary disease; PSI, Pneumonia Severity Index; CURB-65, confusion, urea, respiratory rate, blood pressure, and age 65 years or older.

Exploratory subgroup analyses for day-7 clinical cure are shown in Figure 4 and Supplementary Table S8. The overall unadjusted OR was 2.35 (95% CI 1.23–4.59). Point estimates generally favored adjunctive CHGZ + MXSG across age, sex, COPD, PSI, and CURB-65 strata. However, interaction tests did not identify clear evidence of treatment-effect heterogeneity by age group (*P* = 0.400), sex (*P* = 0.578), COPD status (*P* = 0.568), PSI risk class (*P* = 0.253), or CURB-65 category (*P* = 0.122).

Treatment-emergent safety outcomes are summarized in Table 4. Gastrointestinal adverse events occurred in 12 participants (12.9%) in the CHGZ + MXSG group and 15 participants (16.1%) in the standard-care group (RR 0.80, 95% CI 0.40–1.62; *P* = 0.532). ALT elevation events occurred in 9 participants (9.7%) and 10 participants (10.8%), respectively (RR 0.90, 95% CI 0.38–2.11; *P* = 0.809). Rash occurred in 5 participants (5.4%) in the CHGZ + MXSG group and 4 participants (4.3%) in the standard-care group (RR 1.25, 95% CI 0.35–4.51; *P* = 1.000). Risk differences for these prespecified safety outcomes were small and are provided in Supplementary Table S7.

**TABLE 4:** Treatment-emergent safety outcomes in the full analysis set.

Outcome	CHGZ + MXSG (*n* = 93)	Standard care (*n* = 93)	RR (95% CI)	Descriptive *P*-value
Gastrointestinal adverse event	12 (12.9%)	15 (16.1%)	0.80 (0.40–1.62)	0.532
ALT elevation adverse event	9 (9.7%)	10 (10.8%)	0.90 (0.38–2.11)	0.809
Rash adverse event	5 (5.4%)	4 (4.3%)	1.25 (0.35–4.51)	1.000

Data are presented as n (%). Risk ratios compare CHGZ + MXSG with standard care. Safety comparisons are descriptive because the study was not powered to detect uncommon adverse events. RR, risk ratio; CI, confidence interval; ALT, alanine aminotransferase; CHGZ + MXSG, Chaihu Guizhi Tang combined with Maxing Shigan Tang.

## 4 Discussion

In this prospective non-randomized controlled study of hospitalized older adults with CAP, adjunctive CHGZ + MXSG plus standard care was associated with a higher likelihood of day-7 clinical cure, faster achievement of clinical stability, shorter hospital length of stay, greater early CRP reduction, and greater symptom-score improvement than standard care alone. These associations remained directionally consistent after multivariable adjustment and across several propensity-score approaches. The safety comparison did not show a descriptive excess of gastrointestinal adverse events, ALT elevation events, or rash in the CHGZ + MXSG group. The findings should nevertheless be interpreted with restraint because treatment allocation was not randomized. The consistency of the findings across expanded covariate adjustment, propensity-score approaches, and follow-up sensitivity analyses supports the robustness of the association while not removing the non-randomized nature of the evidence.

The strongest message of the study is not that adjunctive CHGZ + MXSG changed hard outcomes such as mortality, but that it was associated with faster short-term recovery. This distinction matters clinically. CAP guidelines appropriately prioritize antimicrobial selection, severity assessment, and prevention of deterioration (1, 2). For older inpatients who survive the acute episode, however, the time needed to regain stability can affect oxygen use, antibiotic escalation, discharge planning, rehabilitation, and exposure to hospital-related harms. Halm and colleagues established time to clinical stability as a meaningful CAP endpoint because it connects objective bedside signs to management decisions (9). Later studies also showed that reaching stability is influenced by disease severity, host factors, and treatment context (10, 11). In this study, the approximately 0.8-day unadjusted difference in mean time to stability and the adjusted HR of 1.73 suggest a clinically interpretable acceleration of recovery, even if the exact causal contribution of the herbal intervention cannot be isolated fully.

The day-7 clinical cure result is consistent with the time-to-stability finding. A day-7 endpoint is close enough to the acute treatment window to reflect early recovery, yet late enough to capture whether initial improvement is sustained. The unadjusted cure proportion differed by 17.2 percentage points, and the adjusted models gave ORs greater than 2 across conventional and propensity-score analyses. These estimates are not interchangeable with randomized treatment effects, but their consistency argues against the result being driven only by one statistical specification. The lower rate of antibiotic escalation in the CHGZ + MXSG group also supports the possibility of more rapid clinical improvement, although escalation decisions are partly clinician-dependent and may be influenced by local practice patterns.

The CRP findings deserve a careful interpretation. CRP is a nonspecific biomarker, and changes in CRP cannot by themselves prove a mechanism. Still, CRP has long been used as a clinical marker in CAP and has been linked to disease severity, complications, and treatment failure (12–14). Studies of serial CRP suggest that the direction and speed of decline may be informative during follow-up of severe infections (28, 29). In the present study, CRP declined in both groups, which is expected with effective standard care, but the mixed-effects model showed a group-by-time interaction favoring CHGZ + MXSG. This pattern fits the clinical outcomes, but it should be described as supportive evidence of faster inflammatory improvement rather than proof of an anti-inflammatory mechanism.

The broader CAP literature provides a useful context for interpreting an adjunctive therapy signal. Trials of systemic corticosteroids have shown that selected patients with severe CAP or high inflammatory response may benefit from immunomodulation, but these findings have also raised concerns about patient selection, adverse effects, and generalizability (15, 30, 31). The CAPE COD trial reported lower 28-day mortality with hydrocortisone in ICU patients with severe CAP, reinforcing the idea that host inflammatory response can be a therapeutic target in selected settings (16). CHGZ + MXSG is not comparable to corticosteroid therapy in mechanism, intensity, or evidence base. The relevance of this literature is narrower: it supports the principle that recovery from CAP may be influenced not only by antimicrobial therapy but also by the host response. This study adds prospective clinical data for one TCM-based adjunctive strategy in an older inpatient population, while leaving mechanism to future work.

Existing evidence for Chinese medicine in CAP is promising but uneven. A guideline on Chinese patent medicines for CAP highlighted the widespread use of such therapies in China while emphasizing the need for evidence-based selection and better clinical research (17). A systematic review of Maxing Shigan Decoction reported potential benefit when combined with conventional therapy, but the review base was limited by heterogeneity and trial quality concerns (18). A more recent meta-analysis focused on randomized trials of Maxing Shigan Decoction and again suggested possible effectiveness and safety, while also calling for higher-quality evidence (19). A randomized pediatric CAP trial of a related Maxing Ganshi formula reported improvement in symptoms and fever resolution (20). Compared with these studies, the present study focuses on hospitalized older adults, uses clinical stability and CRP trajectories as recovery outcomes, and explicitly addresses non-randomized allocation with propensity-score methods. These features are relevant because older adults with CAP differ from younger or pediatric populations in frailty, comorbidity burden, and vulnerability to adverse events.

Frailty and comorbidity are not background details in older CAP; they are part of the clinical biology of recovery. Reviews and cohort studies have emphasized that CAP in older adults is often shaped by atypical presentation, functional reserve, multimorbidity, and increased risk of adverse outcomes (4, 5). Frailty has been associated with mortality, longer hospital stay, and longer antibiotic use in hospitalized older CAP patients (32). In the present cohort, the groups were similar for several severity indicators, but there were imbalances in frailty, respiratory rate, COPD, congestive heart failure, diabetes, chronic kidney disease, and PCT. These imbalances are exactly why an unadjusted comparison would be inadequate. The adjusted and propensity-score analyses do not remove all bias, but they make the interpretation more credible than a simple two-group comparison. Expanded models incorporating frailty, PSI score, respiratory rate, oxygen saturation, comorbidities, PCT, and pathogen category yielded estimates in the same direction as the prespecified model, suggesting that the primary findings were not solely dependent on the smaller adjustment set.

The use of several propensity-score strategies was intentional. Conventional multivariable adjustment is familiar and efficient, but it can be sensitive to model form in small samples. IPTW aims to create a weighted pseudo-population in which measured baseline covariates are less strongly associated with treatment assignment (24, 25). Trimming reduces the influence of extreme weights, whereas overlap weighting emphasizes participants whose covariate patterns make either treatment strategy plausible (26, 27). In this study, all propensity-score approaches pointed in the same direction for clinical cure, time to stability, and length of stay. That consistency strengthens the robustness of the findings, but it does not eliminate unmeasured confounding, treatment-selection bias, or local practice effects.

The subgroup analysis should not be overread. Point estimates favored CHGZ + MXSG across age, sex, COPD, PSI, and CURB-65 strata, but confidence intervals were wide, and no interaction *P* value suggested a clear subgroup-specific effect. This is not unexpected given the modest sample size and the exploratory nature of the analysis. The larger point estimates observed in lower PSI or CURB-65 strata should be treated as hypothesis-generating only. A future trial would need to prespecify subgroup hypotheses and enroll enough participants to evaluate heterogeneity reliably.

The safety findings are reassuring only within the boundaries of what was measured. Gastrointestinal symptoms, ALT elevation, and rash were similar between groups, and no descriptive excess of these prespecified events emerged. However, the study was not powered to detect rare adverse events, delayed liver injury, herb-drug interactions, uncommon allergic reactions, or safety differences within clinically vulnerable subgroups. More granular adverse-event grading, serious-adverse-event adjudication, discontinuation due to adverse events, serial renal and hepatic laboratory monitoring, and structured concomitant-medication assessment were not fully captured in the analytic dataset. This point is important in older adults, who often receive multiple medications and may have reduced hepatic or renal reserve.

Several limitations should be acknowledged. First, treatment allocation was not randomized and was based on real-world clinical assessment and clinician-patient decision-making, so residual confounding and confounding by indication remain possible despite multivariable adjustment, expanded covariate adjustment, and propensity-score analyses. Second, the open-label design may have introduced observer or performance bias, especially for clinical cure, symptom scores, antibiotic escalation, and discharge-related decisions. Third, although all participants were managed under the same institutional CAP pathway, item-level conventional treatment variables such as antibiotic regimen, time to antibiotics, corticosteroid use, oxygen-therapy modality, bronchodilator use, antiviral treatment, and other supportive measures were not systematically captured for comparative modeling. Fourth, important geriatric factors, including detailed functional status, cognitive impairment, nutritional status, oxygen requirement trajectories, and polypharmacy, were incompletely available. Fifth, the study was modest in size and conducted in a single institutional context, limiting precision, safety assessment, and external generalizability. Sixth, the composite symptom score was an institutional clinical measure and has not been externally validated. Seventh, the study was not prospectively registered in a public trial registry. Finally, the CRP analysis supports a biological signal but does not establish mechanism; no cytokine, microbiome, pharmacokinetic, or pharmacodynamic measurements were included, and the detailed contribution of each component of the combined CHGZ + MXSG regimen cannot be separated in this design.

Despite these limitations, the study has practical strengths. It focused on an older hospitalized CAP population, used clinically interpretable recovery outcomes, reported prespecified safety events, treated the design as non-randomized rather than as a randomized trial, and tested the main findings across several adjustment strategies. These features make the results useful as prospective clinical evidence and as a basis for designing a larger randomized or pragmatic trial. The next step should be a prespecified, adequately powered study with transparent treatment allocation or randomization, standardized dosing and adherence documentation, blinded outcome assessment where feasible, detailed capture of conventional treatment components, and longer safety follow-up.

## 5 Conclusion

Adjunctive CHGZ + MXSG plus standard care was associated with higher day-7 clinical cure, earlier clinical stability, shorter hospital stay, and greater early CRP reduction than standard care alone in hospitalized older adults with CAP. Prespecified safety events were similar between groups. These findings support further evaluation of CHGZ + MXSG as an adjunctive recovery-focused strategy, but they should not be interpreted as definitive causal evidence because treatment allocation was non-randomized and outcome assessment was open-label. Adequately powered randomized or pragmatic trials are needed before clinical efficacy can be established.

## Data Availability

The original contributions presented in the study are included in the article/Supplementary material, further inquiries can be directed to the corresponding author.
