# Supplementary material for: Adjunctive Chaihu Guizhi Tang combined with Maxing Shigan Tang and short-term clinical recovery in hospitalized older adults with community-acquired pneumonia: a prospective non-randomized controlled study
Source: Front Med (Lausanne). 2026 Aug 7;13:1880550. doi: 10.3389/fmed.2026.1880550 (PMC13493553; doi:10.3389/fmed.2026.1880550)
Supplement: Supplementary file 1 [file Table_1.docx]

**Supplementary Material**

**Supplementary Table S1. Participant follow-up and evaluable populations.**

| **Group** | **Enrolled** | **Full analysis population** | **Completed follow-up** | **Incomplete or withdrawn follow-up** | **Completed assigned treatment** | **Per-protocol population** |
| --- | --- | --- | --- | --- | --- | --- |
| CHGZ+MXSG | 93 | 93 | 90 | 3 | 90 | 90 |
| Standard care | 93 | 93 | 89 | 4 | 89 | 89 |
| Total | 186 | 186 | 179 | 7 | 179 | 179 |

The full analysis population included enrolled participants with baseline treatment allocation and day-7 clinical cure data. The per-protocol population included participants who completed the assigned treatment protocol and did not withdraw from follow-up.

**Supplementary Table S2. Data completeness and primary-endpoint precision.**

| **Domain** | **Item** | **CHGZ+MXSG** | **Standard care** | **Status/interpretation** |
| --- | --- | --- | --- | --- |
| Data completeness | Primary clinical cure endpoint | 0/93 (0.0%) | 0/93 (0.0%) | Complete for both groups |
| Data completeness | Major secondary clinical endpoints | 0/93 (0.0%) | 0/93 (0.0%) | Early response, clinical stability, length of stay, escalation, treatment failure, readmission, and mortality |
| Data completeness | CRP at day 5 | 3/93 (3.2%) | 3/93 (3.2%) | Small and balanced missingness |
| Data completeness | CRP at day 7 | 3/93 (3.2%) | 5/93 (5.4%) | Small missingness; handled descriptively in trajectory analysis |
| Data completeness | Symptom score and prespecified safety outcomes | 0/93 (0.0%) | 0/93 (0.0%) | Complete for both groups |
| Endpoint precision | Observed clinical cure rate | 79.6% | 62.4% | Absolute risk difference: 17.2 percentage points |
| Endpoint precision | Primary-endpoint precision |  |  | Approximate power: 73.8%; detectable risk difference for 80% power: 18.4 percentage points |

For repeated laboratory measurements, analyses used available observed measurements and did not impute biomarker values. Percentage values for missingness use the group-specific denominator.

**Supplementary Table S3. Sensitivity analyses for clinical cure at day 7 under alternative follow-up assumptions.**

| **Scenario** | **Risk ratio (95% CI)** | **Risk difference (95% CI)** | **Model effect** | **Model estimate (95% CI)** | **P value** |
| --- | --- | --- | --- | --- | --- |
| Recorded primary outcome | 1.28 (1.06 to 1.54) | 17.2% (4.4% to 30.0%) | OR | 2.35 (1.22 to 4.53) | 0.011 |
| Incomplete follow-up counted as non-cure | 1.35 (1.10 to 1.64) | 20.4% (7.5% to 33.4%) | OR | 2.69 (1.40 to 5.16) | 0.003 |
| Conservative worst-case for CHGZ+MXSG | 1.25 (1.04 to 1.51) | 16.1% (3.4% to 28.9%) | OR | 2.24 (1.16 to 4.33) | 0.016 |
| Best-case for CHGZ+MXSG | 1.40 (1.15 to 1.70) | 23.7% (11.1% to 36.3%) | OR | 3.33 (1.69 to 6.56) | <0.001 |

The recorded day-7 clinical cure endpoint had complete data. Alternative scenarios evaluated whether assumptions about incomplete follow-up or protocol completion changed the direction of the primary endpoint.

**Supplementary Table S4. Multivariable and propensity-score analyses for key clinical outcomes.**

| **Analysis approach** | **Outcome** | **Model/method** | **Effect** | **Estimate (95% CI)** | **P value** |
| --- | --- | --- | --- | --- | --- |
| Multivariable and sensitivity models | Clinical cure at day 7 | Prespecified adjusted model | OR | 2.37 (1.17 to 4.82) | 0.017 |
| Multivariable and sensitivity models | Time to clinical stability | Prespecified adjusted Cox model | HR | 1.73 (1.27 to 2.35) | <0.001 |
| Multivariable and sensitivity models | Hospital length of stay | Prespecified adjusted linear model | β | -0.99 (-1.47 to -0.51) | <0.001 |
| Multivariable and sensitivity models | Clinical cure at day 7 | Expanded geriatric-confounder model | OR | 2.79 (1.26 to 6.21) | 0.012 |
| Multivariable and sensitivity models | Time to clinical stability | Expanded geriatric-confounder Cox model | HR | 1.98 (1.42 to 2.77) | <0.001 |
| Multivariable and sensitivity models | Hospital length of stay | Expanded geriatric-confounder linear model | β | -1.00 (-1.50 to -0.51) | <0.001 |
| Multivariable and sensitivity models | Clinical cure at day 7 | Full baseline-confounder sensitivity model | OR | 3.89 (1.55 to 9.77) | 0.004 |
| Multivariable and sensitivity models | Clinical cure at day 7 | Per-protocol sensitivity analysis | OR | 3.01 (1.42 to 6.36) | 0.004 |
| Propensity-score analyses | Clinical cure at day 7 | Stabilized IPTW | OR | 2.36 (1.18 to 4.75) | 0.016 |
| Propensity-score analyses | Time to clinical stability | Stabilized IPTW | HR | 1.73 (1.27 to 2.34) | <0.001 |
| Propensity-score analyses | Hospital length of stay | Stabilized IPTW | β | -1.08 (-1.64 to -0.51) | <0.001 |
| Propensity-score analyses | Clinical cure at day 7 | Trimmed IPTW | OR | 2.39 (1.19 to 4.79) | 0.014 |
| Propensity-score analyses | Time to clinical stability | Trimmed IPTW | HR | 1.74 (1.29 to 2.36) | <0.001 |
| Propensity-score analyses | Hospital length of stay | Trimmed IPTW | β | -1.08 (-1.65 to -0.52) | <0.001 |
| Propensity-score analyses | Clinical cure at day 7 | Overlap weighting | OR | 2.48 (1.23 to 5.00) | 0.011 |
| Propensity-score analyses | Time to clinical stability | Overlap weighting | HR | 1.78 (1.31 to 2.42) | <0.001 |
| Propensity-score analyses | Hospital length of stay | Overlap weighting | β | -1.07 (-1.63 to -0.52) | <0.001 |
| Propensity-score analyses | Clinical cure at day 7 | 1:1 propensity-score matching | OR | 3.82 (1.66 to 8.77) | 0.002 |
| Propensity-score analyses | Time to clinical stability | 1:1 propensity-score matching | HR | 1.89 (1.33 to 2.69) | <0.001 |
| Propensity-score analyses | Hospital length of stay | 1:1 propensity-score matching | β | -1.11 (-1.74 to -0.48) | <0.001 |

The prespecified model included age, sex, CURB-65 score, COPD, and baseline CRP. The expanded geriatric-confounder model additionally considered BMI, frailty score, PSI score, respiratory rate, baseline SpO2, congestive heart failure, diabetes, chronic kidney disease, PCT, and pathogen category. The full baseline-confounder model further considered smoking, baseline temperature, systolic blood pressure, WBC, neutrophils, BUN, ALT, and center. Propensity-score models used baseline demographic, severity, comorbidity, laboratory, and pathogen-category variables available before treatment allocation.

**Supplementary Table S5. Propensity-score diagnostics and covariate balance.**

| **Method** | **Maximum absolute SMD before adjustment** | **Maximum absolute SMD after adjustment** | **Covariates with SMD ≥0.10 after adjustment** | **Weight distribution** |
| --- | --- | --- | --- | --- |
| Stabilized IPTW | 0.217 | 0.065 | 0 | median 0.88; 1st-99th percentile 0.56-2.82 |
| Trimmed IPTW | 0.217 | 0.058 | 0 | median 0.88; 1st-99th percentile 0.56-2.81 |
| Overlap weighting | 0.215 | 0.000 | 0 | median 0.43; 1st-99th percentile 0.11-0.82 |
| 1:1 propensity-score matching | 0.215 | 0.165 | 4 | Not applicable |

Balance diagnostics summarize baseline covariates. SMD values below 0.10 were considered to indicate acceptable balance. Detailed covariate-level diagnostics were used to generate the Love plot.

**Supplementary Table S6. CRP trajectory and symptom-score component analyses.**

| **Analysis** | **Time point or outcome** | **CHGZ+MXSG** | **Standard care** | **Between-group estimate (95% CI)** | **P value** |
| --- | --- | --- | --- | --- | --- |
| CRP estimated marginal mean, mg/L | Baseline | 94.8 (88.9 to 100.7) | 95.6 (89.7 to 101.5) | -0.8 (-9.2 to 7.6) | 0.851 |
| CRP estimated marginal mean, mg/L | Day 5 | 35.1 (29.2 to 41.1) | 40.8 (34.9 to 46.7) | -5.7 (-14.1 to 2.7) | 0.184 |
| CRP estimated marginal mean, mg/L | Day 7 | 22.9 (17.0 to 28.9) | 31.6 (25.7 to 37.6) | -8.7 (-17.1 to -0.3) | 0.042 |
| CRP trajectory | Group-by-time interaction |  |  | F test from mixed-effects model | 0.001 |
| Symptom-score change from baseline to day 7 | Reduction in cough score from baseline to day 7 | 3.9 ± 1.5 | 3.1 ± 1.3 | 0.81 (0.40 to 1.21) | <0.001 |
| Symptom-score change from baseline to day 7 | Reduction in fatigue score from baseline to day 7 | 3.4 ± 1.3 | 2.9 ± 1.1 | 0.54 (0.18 to 0.89) | 0.003 |
| Symptom-score change from baseline to day 7 | Reduction in composite symptom score from baseline to day 7 | 12.2 ± 2.9 | 11.0 ± 2.8 | 1.26 (0.43 to 2.09) | 0.003 |

CRP estimated marginal means and between-group differences were derived from a mixed-effects model including treatment group, time, group-by-time interaction, and a participant-level random intercept. Symptom-score rows report mean reductions from baseline to day 7; larger reductions indicate greater symptom improvement.

**Supplementary Table S7. Prespecified treatment-emergent safety outcomes.**

| **Safety outcome** | **CHGZ+MXSG** | **Standard care** | **Risk ratio (95% CI)** | **Risk difference (95% CI)** | **P value** |
| --- | --- | --- | --- | --- | --- |
| Gastrointestinal adverse event | 12 (12.9%) | 15 (16.1%) | 0.80 (0.40 to 1.62) | -3.2% (-13.3% to 6.9%) | 0.532 |
| ALT elevation adverse event | 9 (9.7%) | 10 (10.8%) | 0.90 (0.38 to 2.11) | -1.1% (-9.8% to 7.6%) | 0.809 |
| Rash adverse event | 5 (5.4%) | 4 (4.3%) | 1.25 (0.35 to 4.51) | 1.1% (-5.1% to 7.2%) | 1.000 |

Safety comparisons are descriptive because the study was not powered to detect uncommon adverse events.

**Supplementary Table S8. Exploratory subgroup analyses for clinical cure at day 7.**

| **Subgroup category** | **Subgroup** | **n** | **Events in CHGZ+MXSG** | **Events in standard care** | **OR (95% CI)** | **P value** | **P for interaction** |
| --- | --- | --- | --- | --- | --- | --- | --- |
| Age | <75 years | 105 | 39 | 32 | 2.92 (1.22 to 7.01) | 0.016 | 0.400 |
| Age | ≥75 years | 81 | 35 | 26 | 1.65 (0.60 to 4.55) | 0.337 |  |
| Sex | Female | 76 | 31 | 25 | 1.86 (0.66 to 5.25) | 0.241 | 0.578 |
| Sex | Male | 110 | 43 | 33 | 2.72 (1.16 to 6.37) | 0.021 |  |
| COPD | No | 98 | 44 | 31 | 1.85 (0.72 to 4.74) | 0.203 | 0.568 |
| COPD | Yes | 88 | 30 | 27 | 2.72 (1.07 to 6.91) | 0.036 |  |
| PSI risk class | PSI II-III | 77 | 34 | 24 | 3.97 (1.26 to 12.49) | 0.019 | 0.253 |
| PSI risk class | PSI IV-V | 109 | 40 | 34 | 1.76 (0.78 to 3.99) | 0.173 |  |
| CURB-65 score | CURB-65 0-1 | 72 | 31 | 27 | 6.89 (1.41 to 33.56) | 0.017 | 0.122 |
| CURB-65 score | CURB-65 ≥2 | 114 | 43 | 31 | 1.88 (0.86 to 4.09) | 0.113 |  |

Odds ratios and 95% confidence intervals were estimated within each subgroup using logistic regression. P values for interaction were derived from models including treatment-by-subgroup interaction terms. Subgroup analyses were exploratory and were not adjusted for multiplicity.

Model diagnostics. Proportional-hazards diagnostics did not indicate a global violation for the prespecified Cox model (global P=0.202) or the expanded Cox model (global P=0.364). Logistic influence diagnostics for the primary endpoint did not identify a dominant high-influence observation, and linear-model diagnostics for length of stay did not indicate major residual non-normality or heteroscedasticity.
